# Prognostic factors for cancer‐specific survival in 220 patients with breast cancer: A single center experience

**DOI:** 10.1002/cnr2.1675

**Published:** 2022-08-05

**Authors:** Mitra Tarlan, Sedigheh Khazaei, Seyed Hamid Madani, Elaheh Saleh

**Affiliations:** ^1^ Clinical Research Development Center, Imam Reza Hospital Kermanshah University of Medical Sciences Kermanshah Iran; ^2^ Molecular Pathology Research Center Imam Reza Hospital, Kermanshah Universitiy of Medical Science Kermanshah Iran; ^3^ Department of Health Education and Health Promotion, Faculty of Health Semnan University of Medical Science Semnan Iran

**Keywords:** breast cancer, breast cancer prognosis factors, clinical parameters, retrospective analysis

## Abstract

**Objectives:**

Hospital‐based breast cancer survival studies are scarce in western Iran. Furthermore, the relationship between breast cancer survival and clinical parameters has been extensively studied, but many of the findings come from developing countries. This paper aims to estimate the survival of hospital‐based breast cancer patients and its predictor factors.

**Method:**

This retrospective analysis was conducted on 578 patients with primary breast cancer who underwent surgery between 2004 and 2020. Information was collected from medical reports by the Hospital information system in Imam Reza Hospital, Kermanshah, Iran. One‐, 2‐, 5‐, and 10‐year breast cancer‐specific survival has been calculated using the Kaplan–Meier process. Crude and adjusted Hazard Ratios (HR) were calculated using the Cox proportional regression model.

**Result:**

One‐, 2‐, and 5‐year overall breast cancer survival were 219 (99.54%), 196 (89.09%), 159 (72.27%), and 70 (31.81%), respectively. Univariate analysis of breast cancer patients with tumor‐related variables revealed that factors such as age, menopause status, lymph node metastasis, number of lymph nodes, organ metastasis, and stage of disease were significantly associated with disease‐specific survival (*p* < .05). Multivariate analysis demonstrated that metastasis (HR = 41.77, 95% CI: 15.3–114.15) and lymph node metastasis (HR = 5.26, 95% CI: 1.9–14.6) were significantly related to survival.

**Conclusion:**

The findings demonstrate that survival is relatively low and is consistent with late‐stage disease diagnosis. It is believed that this is due to a poor level of awareness, lack of screening programs, and subsequent late access to treatment.

## 1 INTRODUCTION

Breast cancer (BC) is a serious public health issue worldwide. BC affects nearly 1.3 million people worldwide each year, making it the most common type of cancer in women.^1^ According to World Health Organization (WHO) estimates, cancer accounts for approximately one in every six deaths worldwide, with low‐ and middle‐income countries accounting for approximately 70% of cancer deaths. BC with almost two million new cases in 2018, is the most prevalent cancer in women and the second most common cancer in the world after lung cancer.^2^, ^3^

BC accounts for more than 24% of all cancers in Iran, with a prevalence rate of 24.8–34 per 100 000 women and a death rate of less than 10.2 per 100 000 population in 2018.^2^ The long‐term prognosis for women with BC has significantly improved over the last 50 years.^4^ Due to the findings of previous studies,^5^ BC is often detected in Iranian women in an advanced stage; so treatment is delayed.^6^ Abedi investigated the survival rate in Iran and discovered that the rates of survival for 1, 3, 5, and 10 years were 95.8%, 82.4%, 69.5%, and 58.1%, respectively.^7^

More express oncogenes Her 2/neu, pre‐menopausal age, undergoing various forms of treatment (surgery, radiotherapy, chemotherapy, and hormone therapy), Socioeconomic status, fertility status, stage of the disease, the number of lymph nodes involved, type of tumor pathology, higher grade or tumor grade, estrogen and progesterone negativity receptors, and high body mass index (BMI) are relevant factors correlated with lower survival rates.^5^

Western Iran has a population of approximately 4 million and there are relatively few studies relating to exploring the relationship in the Iranian population between these prognostic markers and survival. The identification of the factors contributing to higher survival rates for women with BC will help to identify early diagnosis and improve policies for prevention and treatment, both in the nursing system and in other health fields. Therefore, this study was undertaken to assess the survival rate and probability of mortality in BC patients and to assess the predictive factors for survival.

## 2 MATERIAL AND METHODS

### 2.1 Study population and design

This retrospective analysis was conducted on 578 patients with primary BC who underwent surgery at Imam Reza Hospital in Kermanshah, Iran, between 2004 and 2020. For 234 patients, the survival outcome was not available in the dataset. As a result, they were excluded from the study. In addition, 124 patients were excluded due to exclusion criteria, insufficient medical history, or mortality due to a cause other than BC. In this study, all participants underwent surgery and received radiation, and all except a very limited number received chemotherapy.

### 2.2 Ethical consideration

According to the National Resolution for Human Studies, no informed consent was needed once the present analysis focused on retrospective data collection. Nevertheless, we obtained official permission from the Hospital Majors, labs, municipal, and state Health Secretariats to gain access to databases, laboratory, and medical reports. The research design was approved by the ethical committee and institutional review board of Kermanshah University of Medical Sciences (IR.KUMS.REC.1398.363).

### 2.3 Exclusion criteria

Patients with heart failure, cerebrovascular disease, hematologic illness, any inflammatory symptoms or conditions, peripheral arterial disease, coronary artery disease, end‐stage renal disease, peripheral arterial disease, or a lack of pathologic or laboratory data were excluded.

### 2.4 Collection data

Medical records for the Hospital Information system (HIS) were reviewed and age, menopause status, lymph node metastasis, diameter and side of the tumor, stage of disease, pathological tumor types, Oral Contraceptive Pill (OCP) consumption, family history, number of babies, breastfeeding status, estrogen receptor (ER), progesterone receptor (PR), human epidermal growth factor receptor‐2 (HER‐2) levels were collected as dependent variables. BC overall survival (independent variables) was calculated from the date of operation to the date of death for women whose death was specifically due to cancer. Immunohistochemical (IHC) staining in tumor tissue was determined by HER‐2 levels: IHC 3+ and IHC 2+ were classified as HER‐2 positive tumors; IHC 1+ or IHC‐ were classified as HER‐2 negative tumors. Her‐2 status was determined using IHC staining and those with 3+ results assumed positive, 2+ was indeterminate and 1+ assumed negative.

Luminal‐A is defined as ER+ and/or PR+ / HER2‐, Luminal‐B is ER+ and/or PR+/HER2+, HER2 overexpressed is ER‐/PR‐/HER2+, and the triple negative is ER‐/PR‐/HER2‐. For BC, the morphological and topographic classifications used were ICD‐O^15^ and ICD‐10^16^, respectively.^8^

All patients with a histopathologic diagnosis of cancer during the study period (ICD‐10: C50 and D05.1) corresponding to malignant clinical tumors, primary site (ICD‐O: code 3), and malignant metastatic site (ICD‐O: code 6) were included. All cases were diagnosed pathologically by biopsy or surgical specimens.

Distant Metastases have been identified through radiological analysis, such as magnetic resonance imaging, ultrasound, bone scintigraphy, and computed tomography.

### 2.5 Following‐up investigation

About 578 cases of these women underwent follow‐up investigations for 60–192 months following BC surgery.

### 2.6 Statistical analysis

SPSS version 13.0 software was used for statistical analysis. Survival curves are drawnusing Life‐tables and survival rate comparisons were done by using the Chi‐squared test. Cox regression model analysis and multivariate analysis were performed to determine prognostic factors. A *p*‐value of less than .05 was considered statistically significant. The study endpoints were 5‐ and 10‐year disease‐free survival.

## 3 RESULTS

Of the 220 studied patients, 81 (36.8) died by the end of the analysis. The age of patients ranged from 20 to 83 years with a mean of 47.48 ± 11.33 and a median of 46 years.

**FIGURE 1:**
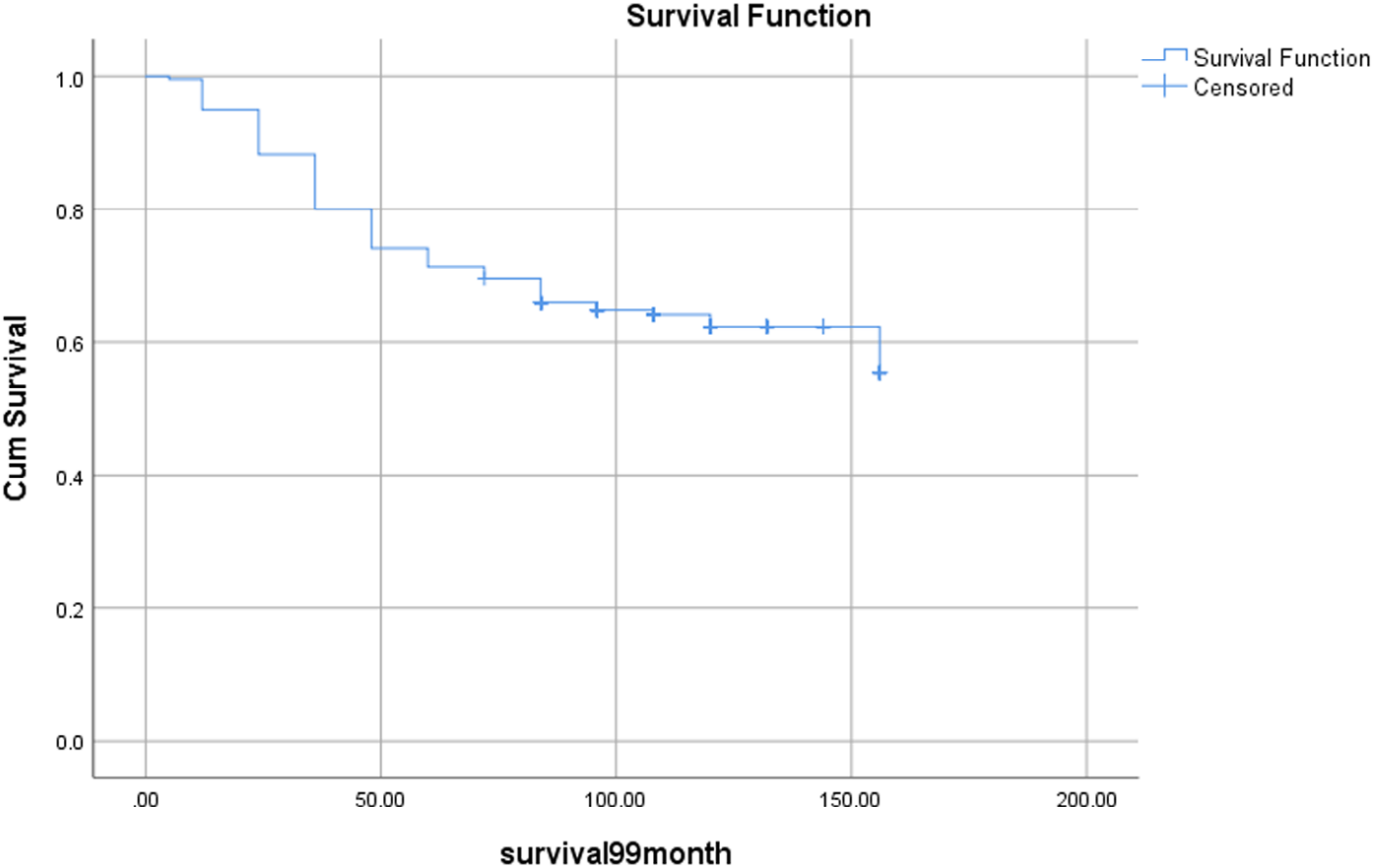
The plot of overall survival for patients with breast cancer

The overall survival rates of 1‐, 3‐, 5‐, and 10‐year for the 220 cases were 95%, 80%, 71%, and 62%, respectively, with a median survival period of 96 months (Figure 1).

Table 1 provides an analysis of tumor‐related factors and prognosis for 220 women with BC. The highest number of patients was in the age group of 20–50 (predominantly in the age group of 40–50). In addition, 35.9% of patients were in the early stage of diagnosis. The 1‐, 3‐, 5‐, and 10‐year survival was inversely related to the stage at diagnosis (Table 1).

**TABLE 1:** Clinical, pathological, and biological characteristics of patients and univariate analysis

Variable	*N* (%)	Mean ± SE		Years				*p*‐value
				Number of survivors				
			Median OS (month)	1	3	5	10	
Survival	220	115.16 ± 3.78	96	219	196	159	70	
Age (year)								
20–50	138 (63.3%)	112.11 ± 4.35^a^	96	138	126	108	48	.01*
50–60	48 (22%)	113.42 ± 8.96^a^	96	48	41	33	13	
60–70	19 (8.7%)	91.5 ± 13.36^b^	84	19	16	12	6	
70–95	13 (6%)	76.08 ± 17.07^b^	48	13	12	6	3	
Marital status								
Single	14 (6.5%)	100.64 ± 13.9	102	13	13	9	7	.644
Married	203 (93.5%)	115.70 ± 3.92	96	203	181	148	62	
Menopause								
Premenopause	145 (66.8%)	121.68 ± 4.39	96	144	132	112	50	.012*
Postmenopause	72 (33.2%)	102.02 ± 7.06	84	72	61	45	20	
Lump node metastasis								
Yes	79 (56.8%)	102.36 ± 6.72	84	78	69	49	24	<.001*
No	60 (43.2%)	145.80 ± 3.46	120	60	60	58	31	
Lymph node number								
Less than 4 or 4	39 (54.2%)	19.07 ± 9.41^a^	108	39	35	30	16	.002*
5–9	22 (30.6%)	93.27 ± 11.06^a^	84	22	20	12	6	
10 or more than 10	11 (15.3%)	56.09 ± 10.76^b^	48	10	8	3	1	
Metastasis								
Yes	75 (35%)	53.18 ± 4.54	36	74	53	24	3	<.001*
No	139 (65%)	149.60 ± 2.24	108	139	137	132	65	
Organ metastasis								
OCP before menopause								
Yes	150 (70.1%)	121.03 ± 4.23	96	149	134	116	48	.066
No	64 (29.9%)	105.27 ± 7.21	84	64	58	41	20	
FH family history								
Yes	99 (46.3%)	117.47 ± 5.61	96	98	89	71	26	.642
No	115 (53.7%)	115.27 ± 5.19	96	115	102	85	42	
Number of children								
Less than 2 or 2	56 (25.9%)	124.33 ± 6.83	96	56	52	44	16	.134
More than 2 and less than 5	84 (38.9%)	116.58 ± 6.00	102	84	75	61	30	
Or more than 5	55 (25.5%)	109.09 ± 7.85	84	55	49	39	16	
Without children	21 (9.7%)	101.38 ± 12.04	84	20	18	13	7	
Breast feeding								
Yes	185 (86.4%)	117.76 ± 4.03	96	185	168	138	58	.32
No	29 (13.6%)	104.97 ± 10.91	84	29	25	18	10	
Side of tumor LR								
L	92 (46.2%)	114.45 ± 5.87	96	92	82	67	33	.977
R	105 (52.8%)	114.65 ± 5.59	96	104	93	74	30	
Size of tumor								
2 or less than 2	35 (22%)	112.58 ± 8.88	108	35	32	27	13	.448
More than 2	124 (78%)	114.27 ± 5.10	96	123	111	89	43	
Stage								
0–1	52 (35.9%)	147 ± 3.91^a^	108	52	52	50	22	<.001
2a	6 (401%)	129 ± 2.59^a^	120	6	6	6	4	
2b‐4	87 (60%)	84.16 ± 6.46^b^	60	86	68	43	18	
Unknown								
Histology								
Ductal in situ	3 (1.4%)	120 ± 19.59	108	3	3	3	1	.193
Ductal invasive	212 (96.8%)	115.48 ± 3.86	96	211	189	153	68	
Others	4 (1.8%)	66 ± 16.15	66	4	3	2	69	
ER								
Positive	136 (61.8%)	118.89 ± 4.66	96	136	125	101	48	.286
Negative	84 (38.2%)	109.24 ± 6.38	96	83	71	58	22	
PR								
Positive	135 (61.4%)	120.82 ± 4.57	96	134	125	103	49	.103
Negative	85 (38.6%)	106.23 ± 6.47	84	85	71	56	21	
Her 2								
Positive	116 (53.7%)	112.54 ± 5.25	96	115	103	81	37	.456
Negative	100 (46.3%)	118.28 ± 5.60	96	100	89	74	31	
P53								
Positive	52 (36.6%)	129.92 ± 6.71	108	1	1	42	20	.228
Negative	89 (62.7%)	115.38 ± 6.14	96	52	49	64	26	
Ki67								
Positive	86 (92.5%)	122.88 ± 5.94	96	86	77	66	21	.317
Negative	7 (7.5%)	140.57 ± 14.28	132	7	7	6	4	
Type of breast								
Luminar A	71 (32.9%)	124.41 ± 6.31	108	71	66	55	23	.215
Luminar B	87 (40.3%)	104.63 ± 5.43	96	86	78	60	29	
Her2 overexpressed	30 (13.9%)	109.06 ± 10.71	96	30	26	20	7	
Triple‐negative	28 (13%)	109.71 ± 11.22	90	28	22	20	9	
Nuclear grading (NG)								
1/3	26 (21.7%)	123.367 ± 7.16	9	26	25	23	9	.145
2/3	70 (58.3%)	117.56 ± 6.86	84	70	61	50	15	
3/3	24 (20%)	104.29 ± 12.16	102	23	20	15	10	
Margin grading (MG)								
1/3	19 (14.7%)	123.36 ± 7.16	84	19	17	13	2	.451
2/3	88 (68.2%)	117.56 ± 6.86	96	88	77	64	29	
3/3	22 (17.1%)	104.29 ± 12.16	102	22	21	16	7	
Vascular invasion								
Positive	95 (75.4%)	115.23 ± 5.77	96	94	84	68	28	.742
Negative	31 (24.6%)	112.13 ± 10.63	96	31	27	22	11	
Skin involvement								
Positive	4 (6.3%)	87.00 ± 22.60	84	4	4	2	2	.463
Negative	69 (93.7%)	122.49 ± 6.98	108	59	55	45	29	
Nipple involvement								
Positive	3 (4.3%)	92.00 ± 32.66	84	3	2	2	1	.805
Negative	66 (95.7%)	118.64 ± 6.9	114	66	59	49	33	
Margin involvement								
Positive	8 (14%)	94.5 ± 17.42	84	8	7	5	2	.805
Negative	49 (86%)	122.57 ± 7.39	108	49	45	39	20	

Dissimilar letter indicate a significant difference between groups (*p < 0.05).

Univariate analyses of BC patients with tumor‐related factors indicated that factors such as age, menopause status, lymph node metastasis, number of involved lymph nodes, distant metastasis, and stage significantly correlated with disease‐specific survival rates (*p* < .05) (Table 1).

Because of the correlation between the lymph node number and lymph node metastasis, we only included the variables metastasis, lymph node metastasis, age, and menopause in the Cox model. In this model, only the variables of metastasis (HR = 41.77, 95% CI: 15.3–114.15) and metastasis in lymph nodes (HR = 5.26, 95% CI: 1.9–14.6) are significant. According to the Cox model, the risk of death in BC for patients with metastasis is 41.71 times higher than those without metastasis, and the risk of death in people with lymph node metastasis is 5.26 times higher than those without lymph node metastasis (Table 2).

**TABLE 2:** Multivariate cox regression analysis

Variable	Standard error (*β*)	)WALD)	*p* value	HRa	95% CI
Age (year)					
20–50*				1	
50–60	−1.26 (1.04)	1.47	.23	0.28	(0.04, 2.18)
60–70	−0.396 (1.24)	0.102	.75	0.67	(0.06, 7.6)
70–95	−0.405 (1.18)	0.117	.73	0.67	(0.07, 6.8)
Premenapose*				1	
Postmenapose	0.704 (1.07)	0.431	.51	2.02	(0.25, 16.54)
Lump node metastasis					
No*				1	
Yes	1.7 (0.52)	10.18	<.001	5.26	(1.9, 14.6)
Metastasis					
No*				1	
Yes	3.73 (0.513)	52.94	<.001	41.77	(15.3, 114.15)

* *p* < 0.05.

**FIGURE 2:**
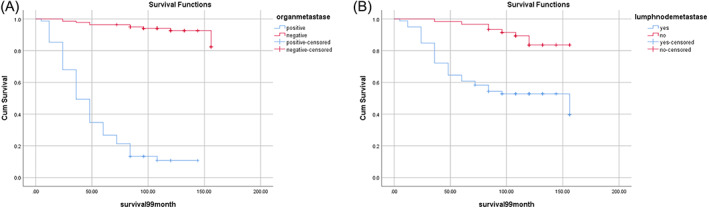
Factors related to survival rates of breast cancer patients. (A) Relationship between organ metastasis and disease‐specific survival rates. (B) Relationship between lymph‐node metastasis and survival rates

There was a significant negative correlation between metastasis and axillary lymph node metastasis and long‐term disease‐free survival rates, as seen in Figure 2. Increased involved lymph node numbers were correlated with higher mortality rates.

## 4 DISCUSSION

Cancer is the world's second leading cause of death, accounting for almost 9.6 million deaths in 2018.^2^ Epidemiological evidence showed that the largest prevalence of BC was among the 50‐ and 60‐year age group, followed by the 40‐ to 49‐year age group. We demonstrated that the largest prevalence of BC was in the age group of 20–50 (predominantly in the age group of 40–50).^9^, ^10^ This then reinforces the need to do mammography in the age group of 40–50. Various studies conducted in Iran have estimated the mean age at the time of diagnosis to be 45–50 years.^11^ The mean age of patients at the time of diagnosis in the present study was about 47.71 years, which is consistent with the findings of other studies.^12^, ^13^ However, these results do not agree with the results of studies in Western Europe and North America.^14^, ^15^ This could be due to the delay in the diagnosis of cancer in Iran, which has been mentioned in other studies.^11^, ^16^ Two other studies also reported that mean age affects the survival of BC.^17^, ^18^

Factors influencing the survival rate of women with BC have become extremely important for patients. Several risk factors related to BC have been reported by Roder et al.,^19^ including tumor size, higher grade, positive nodal status, ER‐negative status, vascular invasion, and multifocality. Menopausal status, tumor size, axillary lymph node metastasis, and TNM stage were found to be associated with a 5‐year overall survival rate in 246 BC patients.^20^

Findings of this study showed that metastasis and metastasis in lymph nodes were the most important factors that significantly reduced the survival of the subjects, which is consistent with the results of other studies conducted in Iran.^12^, ^21^

In the univariate test, the disease stage, menopausal status, age, and number of lymph nodes involved all showed a significant relationship with decreased survival.

In the analysis of the marital status, 93.5% of the patients were married, but no significant relationship was shown between married and single women and survival rate. In another study in Kermanshah, women who had never been married, had a higher chance of developing BC than married, divorced, and widowed women. However, the observed relationship was not statistically significant.^22^

A meta‐analysis review in Iran estimated the 1‐, 2‐, 3‐, 5‐, and 10‐year BC survival rates in Iran to be 95.8%, 82.4%, 69.5%, and 58.1%, respectively, in line with the findings of this study. In general, since this study was not based on data from the national or provincial registration program, it did not make sense to judge whether the observed survival rate increased or decreased. In addition, the generalization of results to BC patients in the country should be done with great caution. The 5‐year overall survival rate in Iran compared to 84% in the United Kingdom,^23^ 89% in the United States,^24^ 71% in Germany,^25^ 65% in Greece,^26^ 64% in Oman^27^ and 46% in India^28^ suggesting that Iran's survival is significantly less than that of European countries and the United States, which is in line with the result of Vahdaninia et al. study.^21^ There are several possible explanations for this. Women's knowledge of BC in Iran is limited—Iranian women know little to nothing about breast self‐examination and its impact on early diagnosis and prognosis.

In addition, the difference in the survival rate of BC between countries and regions of the same country, however, persists and is not easy to explain. In one country versus another, the highest survival rate could be due to the availability of better treatment or similar treatment becoming more effective when the diagnosis is made at an earlier stage of the disease.^24^

In BC, the presence of lymph node metastasis is a predictive factor for women's disease survival and recurrence, reducing survival by 40% relative to women with non‐metastatic disease survival.^29^ This research showed that a greater percentage of positive axillary lymph nodes raised the risk of death associated with BC. This was consistent with another study, which showed that axillary lymph node metastasis was the most significant prognostic factor influencing local regulation, disease‐free survival, and overall survival.^30^ According to Akbari et al.,^31^ the most important factor influencing patient survival is axillary lymph node involvement, as patients with involved lymph nodes lived shorter than those without involved lymph node involvement. If the disease is diagnosed at an early stage where the lymph nodes are not involved and the patients are treated correctly, they will survive longer. In this study, lymph node involvement increases the death risk by a factor of 5.26.

The number of nodes involved was the most important multi‐factorial research predictor of survival. According to our results, not only did node‐positive patients have a shorter survival rate compared to node‐negative patients, but the risk of mortality also increased as the number of nodes involved increased.^32^ Compared to non‐metastatic BC, metastatic breast cancer (MBC) is commonly considered to have a low prognosis. Common sites of distant metastasis in BC include bone, liver, lung, and brain.^33^ Previous studies have shown that in BC patients, the bone is the most common distant metastatic organ.^34^, ^35^ Similarly, our findings also indicate that the most prevalent subgroup in the study population is bone and liver metastasis, accounting for 21.3% of the total patients.

Dabakuyo et al.^36^ reported that the survival rate was lower in patients above 60 years of age or postmenopausal patients. The cancer mortality rate has also been reported to have risen with age in Northern Vietnam. According to the results of this study, postmenopausal patients were shown to have a significantly lower specific survival rate than premenopausal patients.^37^ Contrary to the results of this study, D'Eredita et.al^38^ demonstrated that tumor size was significantly associated with BC survival, as patients with tumor size of 5 cm and more had a higher chance of death than those with tumor sizes of 2 cm and less. This finding was consistent with two other studies.^12^

Similar to other studies, we found that the stage of the disease is one of the most important factors in the survival rate of patients with BC. The late stage of diagnosis was separately related to lower survival rates globally.^4^, ^39^, ^40^, ^41^ Of the predictive factors, the absence of metastatic lymph node involvement and the expression of estrogen receptors are the most important for a better prognosis. In the early stages, this enhances the value of detection and care, increasing the chance that the disease has not yet reached the axillary lymph nodes, which improves recovery, cure prospects, and enables less aggressive therapies to be implemented.^10^, ^30^, ^42^ In contrast to lymph node involvement, we found no significant relationship between estrogen receptor status and survival, which may be due to the small sample size in our study.

Since breast‐conserving surgery has become the treatment of choice for early‐stage BC patients, the function of surgical margins is poorly known and is still under discussion.^43^, ^44^ Fujimoto and colleagues demonstrated that women with positive margins showed poorer 2‐ and 5‐year BC survival compared to women with negative margins. In our study, no significant relationship was found between margin involvement and survival rate. Skin and nipple involvement and vascular invasion were not associated with survival, which is consistent with the result of Rezaianzadeh et al.^12^

This research showed no indications of a relationship between BC family history and survival and found that tumor features are identical in patients with a positive and negative family history. This is in line with the findings of several other studies.^45^, ^46^, ^47^

Contrary to the results of this study, Akbari et al.^31^ and Fallahzadeh et al.^48^ found that survival is impacted by the side of the tumor (left or right breast) and that more patients survived with a tumor in their left breast than those with a tumor in their right breast. The study also found no association between histological type and survival in common with other works.^49^, ^50^

Lööf‐Johanson et al.^51^ reported that there was a higher survival rate for women with primary BC if overall breastfeeding was longer than 6 months and/or if they had at least one pregnancy. The results of our study were in line with other studies that reported different results.^52^, ^53^, ^54^ The findings of previous research on OCPs and BC mortality among women were inconsistent; some reported lower mortality among OC Ps users^55^, ^56^ and some recorded higher mortality^57^, ^58^, ^59^ or no effect.^60^, ^61^, ^62^

The prevalence of TNBC in this research is in line with the literature, as it accounts for 10% to 20% of all BCs.^63^, ^64^ Patients with TNBC have been found to have a poor prognosis.^65^ On the other hand, another analysis found that there were no significant differences in 3‐year survival between TNBC cancers and other BCs.^66^ Further studies have found that the difference between the TNBC and non‐TNBC groups peaked at 3 years, but this difference has declined with time, indicating that long‐term survivors (over 10 years) in the TNBC group may have survival comparable to cases of non‐TNBC.^67^, ^68^ While there is no difference in OS between patients with TNBC and non‐TNBC in this research, maybe a greater number of cases between the two groups would have yielded different outcomes.

There are certain limitations in our analysis. Details on some of the explanatory variables are missing; some patients were not available after 10 years. Information on ER, PR, and HER2, vascular invasion, and nipple and skin involvement was absent in nearly half of the initial sample population.

## 5 CONCLUSION

In conclusion, the findings presented in this study indicate a comparatively poor overall survival rate of 5 and 10 years for women diagnosed with BC in Iran. Qualitative information on BC patients and health care providers recognized that a healthier lifestyle and the value of early diagnosis should be obtained to improve their awareness, and also study educational programs should be provided for the target population to prevent deaths related to BC. The findings of the review of 26 explanatory factors presented in this report indicate that the survival of BC in western Iran is impaired by delaying diagnosis and, therefore, by late‐stage disease. We conclude that this is due to a poor level of awareness, cultural differences, and lower access to treatment. Further study is required in Iranian women to test these hypotheses and thus formulate effective approaches to effectively boost survival.

## AUTHOR CONTRIBUTIONS

*Data curation*, M.T.; *Investigation*, M.T., S.K., E.S.; *Methodology*, M.T., S.H.M.; *Writing – Original Draft*, M.T.; *Writing – Review and Editing*, M.T., S.H.M., E.S.; *Formal Analysis*, S.K., E.S.; *Visualization*, S.K.; *Conceptualization*, S.H.M.; *Supervision*, S.H.M.

## CONFLICT OF INTEREST

The authors have stated explicitly that there are no conflicts of interest in connection with this article.

## Data Availability

All data required to assess the research's conclusions are included in the article and/or the supplemental material.
